# Stimulatory Effect of Indolic Hormone on As_2_O_3_ Cytotoxicity in Breast Cancer Cells: NF-κB-dependent Mechanism of Action of Melatonin

**DOI:** 10.22088/IJMCM.BUMS.7.3.158

**Published:** 2018-10-14

**Authors:** Ava Safaroghli- Azar, Atieh Pourbagheri- Sigaroodi, Davood Bashash, Elaheh Nooshinfar, Ali Anjam-Najmedini, Soroush Sadeghi, Mostafa Rezaie-Tavirani, Mohammad Esmaeil Akbari

**Affiliations:** 1 *Student Research Committee, Department of Hematology and Blood Banking, School of Allied Medical Sciences, Shahid Beheshti University of Medical Sciences, Tehran, Iran.*; 2 *Department of Biotechnology, Faculty of Advanced Sciences and Technology, Pharmaceutical Sciences Branch, Islamic Azad University (IAUPS), Tehran, Iran.*; 3 *Department of Hematology and Blood banking, School of Allied Medical Sciences, Shahid Beheshti University of Medical Sciences, Tehran, Iran.*; 4 *Cancer Research Center, Shohada Hospital, Shahid Beheshti University of Medical Sciences, Tehran, Iran.*; 5 *Proteomics Research Center, Faculty of Paramedical Sciences, Shahid Beheshti University of Medical Sciences, Tehran, Iran.*

**Keywords:** As_2_O_3_, apoptosis, combination therapy, melatonin, NF-κB

## Abstract

The advent of combination therapy unprecedentedly shifted the paradigm of cancer treatment by reconstructing the conventional protocols. By identifying the anti-tumoral activity for different natural products, recent interest has focused on inventing the combined- modality strategies to increase the cure rates of cancer, while reducing the toxic side effects of current intensive regimens. To evaluate whether melatonin, indolic hormone produced mainly by the pineal gland, could enhance the pro-apoptotic effect of arsenic trioxide (As_2_O_3_) in breast cancer, MCF-7 cells were treated with As_2_O_3_-plus- melatonin and then the survival, proliferative rate, caspase-3 activity, and mRNA expression level of anti- apoptosis target genes of NF-κB were investigated. Our results delineated that exposure of MCF-7 cells to As_2_O_3_ not only reduced the survival of the cells, but also induced a caspsase-3-dependent apoptotic cell death. Noteworthy, an enhanced induction of apoptosis was found using As_2_O_3_ in combination with melatonin. Moreover, RQ-PCR analysis revealed that the enhanced cytotoxic effect of As_2_O_3_ in the presence of melatonin is mediated, at least partly, through suppressing the expression of NF-κB anti-apoptotic target genes such as *MCL-1*, BCL-2, survivin, *XIAP*, and *c-IAP1* in breast cancer cells. The resulting data showed that As_2_O_3_, either alone or in combination with melatonin, exerted significant cytotoxic effect against MCF-7 cells. However, further investigations are needed to provide valuable clues for expediting this combination as a therapeutic strategy for breast cancer.

The therapeutic approaches in breast cancer, as the most common malignancy in womankind in term of morbidity and mortality (1), still remain an unsolvable dilemma; therefore, the entrance of the novel anti-tumoral agents in combination with chemotherapy or radiotherapy turns to be groundbreaking for more efficient treatment. During the last decade, identifying the potent anti-cancerous property of arsenic trioxide (As_2_O_3_) revitalizes the popularity of this agent as an effective chemotherapeutic drug not only for hematologic malignancies but also for solid tumors (2). An overwhelming number of studies imply that As_2_O_3 _exerts its variegated apoptotic effects through various mechanisms including modulation of the intracellular glutathione redox system (3), induction of mitotic arrest (4), DNA damage and inhibition of DNA repair (5). Despite its prodigious anti- tumoral activities, the clinical use of this agent became restricted due to the toxic side effects of long-term intake of high doses of As_2_O_3_. A recent renaissance of As_2_O_3_ following the novel disclosure indicating that As_2_O_3_ is an ideal agent to be used in combination therapy, has revived the interest in this ancient drug and has constructed its future direction into clinical investigations.

Melatonin, the master biologic clock neurohormone, is basically considered as the main regulator of circadian rhythm and sleep quality (6). Thus far, a plethora of biological actions have been reported for melatonin; however, recent investigations concerning its inhibitory impact on the development, promotion, and progression of various types of cancers, in particular, breast cancer have attracted tremendous attention (7). In the last decades, several experimental studies have outlined that melatonin not only possess a chemo-preventive property (8), but also exerts a pro-apoptotic activity in cancer cells mostly through generation of intercellular ROS (9, 10). For the nonce, multiple published reports have discussed about the intensifying effect of melatonin in combination with different chemotherapeutic drugs, especially DNA damaging agents such as cis-platin (11), cyclophosphamide (12), and doxorubicin (13). Based on the convergence mechanisms between melatonin and As_2_O_3 _(14, 15), it was tempting to evaluate whether melatonin could enhance the pro-apoptotic effect of As_2_O_3 _in MCF-7 breast cancer cells.

## Materials and methods

**Cell line and reagents**

The human hormone-sensitive breast adenocarcinoma cell line MCF-7 (Pasteur Institute, Tehran, Iran) was maintained in Dulbecco’s modified Eagle’s culture medium (DMEM) supplemented with 10% fetal bovine serum, 2 mM L-glutamine, 100 U/mL penicillin and 100 µg/mL streptomycin (Gibco, Life Technologies, Carlsbad, CA). Cells in exponential growth phase were used for experiments. DMEM medium was renewed every 3 days and the cells were harvested with 0.05% trypsin on the sixth day after seeding. The common chemotherapeutic drug, As_2_O_3_, was purchased from Sina Darou (Tehran, Iran). A stock solution of As_2_O_3_ was dissolved in culture medium (DMEM) to attain the concentrations of 1-5 µM. For extensive experiments, MCF-7 cells were also treated with desired concentrations of an indolamine hormone, melatonin, either alone or in combination with As_2_O_3_. In all experiments, equal amount of solvent, ethanol, was added in medium as controls at the final concentration of 0.1%. All drug treatments were carried out in three independent experiments and all assays were performed in triplicate.

**Trypan blue exclusion assay**

Breast cancer-derived MCF-7 cells were seeded at the density of 450 × 10^5^ cells and were incubated with various concentrations of As_2_O_3 _and melatonin, either alone or in combined modality. After 48 h, drug treated-cells were trypsinized, centrifuged and the cell pellets were re-suspended in phosphate-buffered saline (PBS) in combination with 0.4 % trypan blue (Invitrogen, New Zealand). By using a Neubauer hemocytometer, the number of viable cells was counted and afterwards the percentage of viable cells was assessed using the following formula: viability (%)= viable cell count/total cell count×100.

**MTT assay**

Microculture tetrazolium assay (MTT) was applied to explore the impeding effect of As_2_O_3_, either as a single agent or in combination with melatonin, on the ability of breast cancer cells to metabolize thiazolyl blue tetrazolium bromide into formazan crystals. After incubation of cells with agents up to 48 h, cells were incubated with 100 μl of MTT (0.5 mg/ml) solution for further 3 h in a humidified incubator. The optical densitometry (OD) of a resulting formazan solubilized with dimethyl sulfoxide was measured in an enzyme-linked immunosorbent assay (ELISA) reader (BioTek® Instruments, Inc., USA) at the wavelength of 570 nm.

**BrdU cell proliferation assay**

The effect of As_2_O_3 _on the proliferative capacity of MCF-7 cells was assessed using bromodeoxyuridine (BrdU) kit (Roche, Germany). Initially, MCF-7 cells were seeded into 6-well plates overnight and then were treated with As_2_O_3_ for the next 48 h. In the final 12 h of the desired incubation times, 10 µl of BrdU labeling solution was added. For cell fixation and denaturation of DNA, 200 μl of FixDenat solution was added to each well. After 30 min and discarding FixDenat, 100 μl peroxidase-conjugated anti-BrdU (anti-BrdU-POD) antibody was added to each well. Finally, the cells were exposed to 100 μl of substrate tetramethyl-benzidine (TMB) for 3 min at room temperature and the reaction product was quantified by measuring the absorbance at 450 nm in an ELISA reader.

**Median-effect analysis of drug combinations**

To evaluate whether there was a synergistic

effect between As_2_O_3_ and melatonin, we computed the combination index (CI) using the method developed by Chou and Talalay (16). The dose which may be reduced in a combination for a given level of effect as compared to the concentration of individual drug alone was defined as dose reduction index (DRI) and calculated as follow: (DRI)1 = (Dx)1/(D)1 and (DRI)2 =(Dx)2/(D)2, where (Dx)1 and (Dx)2 indicate the individual dose of As_2_O_3_ and melatonin required to inhibit a given level of viability index, respectively. (D)1 and (D)2 are the doses of As_2_O_3_ and melatonin necessary to produce the same effect in combination, respectively.

**Phosphatidylserine (PS) externalization (annexin- V assay)**

Annexin-V/PI staining was applied to investigate whether As_2_O_3_ as a single agent or in combination with melatonin could induce apoptotic cell death in breast cancer cells. After 48 h incubation of MCF-7 cells with designated concentrations of As_2_O_3_ and melatonin, the trypsinized adherent cells were centrifuged and then were washed twice with cold PBS. Cells were re-suspended in 1X Binding Buffer at a concentration of 1×10^6^ cells/ml. In the next step, annexin-V-Flous and PI stain were added to each sample, the cell suspension was mixed gently and incubated for 20 min in the dark. Finally, 400 µl binding buffer was added to each sample and the amount of externalization of phosphatidylserine was analyzed by flow cytometry.

**Caspase -3 activity assay**

The caspase-3 assay (Sigma, USA) is based on spectrophotometric detection of the p-nitroaniline (pNA) after the hydrolysis of the peptide substrate acetyl-Asp-Glu-ValAsp p-nitroanilide (Ac-DEVD-pNA) by caspase 3. For evaluating whether As_2_O_3_ and melatonin could reduce the survival rate of MCF-7 cells through activation of caspase-3, breast cancer cells were treated with each agent, either alone or in combined modality. Following 48 h treatment, the trypsinized adherent cells were centrifuged at 600 x g for 5 min and washed with 1 ml PBS. After gently discarding the supernatant, the cell pellets were re-suspended in lysis buffer at the concentrations of 1×10^7^ cells/100 µl and were incubated on ice for 20 min. Next, the lysed cells were centrifuged at 20000 x g for 10 min. Next, 5 μg of the supernatant was added to 100 µl solution consisting of 85 μl assay buffer and 10 μl caspase-3 substrate acetyl-Asp-Glu-Val-Asp pnitroanilide. The concentration of the pNA released from the substrate was quantified spectrophotometrically from the absorbance values at 405 nm.

**Quantitative real-time PCR**

To perform qRT-PCR, the cells were treated with As_2_O_3_ and As_2_O_3_-plus-melatonin, and then RNA was extracted by high pure RNA isolation kit (Roche, Germany) and measured by Nanodrop ND-1000 (Nanodrop Technologies, Wilmington, Delaware, USA).1 µg of RNA from each sample was used for reverse transcription using the revertAid First Strand cDNA synthesis kit (Takara BIO, Japan). The synthesized cDNA was used to carry out qRT-PCR using SYBR Premix Ex Taq technology (Takara BIO, Japan) on a light cycler instrument (Roche, Germany). Table 1 summarizes the nucleotide sequences of the primers used in qRT-PCR analysis. The alteration in the mRNA expression level of NF-κB anti-apoptotic target genes, such as myeloid cell leukemia-1 (*MCL-1*), B cell lymphoma-2 (*BCL-2*), survivin, X-linked inhibitor of apoptosis protein (*XIAP*), and cellular inhibitor of apoptosis protein 1 (*c-IAP1*) were analyzed in comparison with the expression of *HPRT*, as the housekeeping gene.

**Statistical analysis**

Data are expressed as the mean ± SD of three independent experiments. The significance of differences between experimental variables was determined by the use of two-tailed Student’s t-test and by one-way variance analysis. To compare the control group and the drugs- treated cells, the Dunnett’s multiple comparison test was used. A probability level of P<0.05 was considered statistically significant.

## Results

**Oncocytic effect of As2O3 on MCF-7 breast cancer cells**

**Fig. 1:**
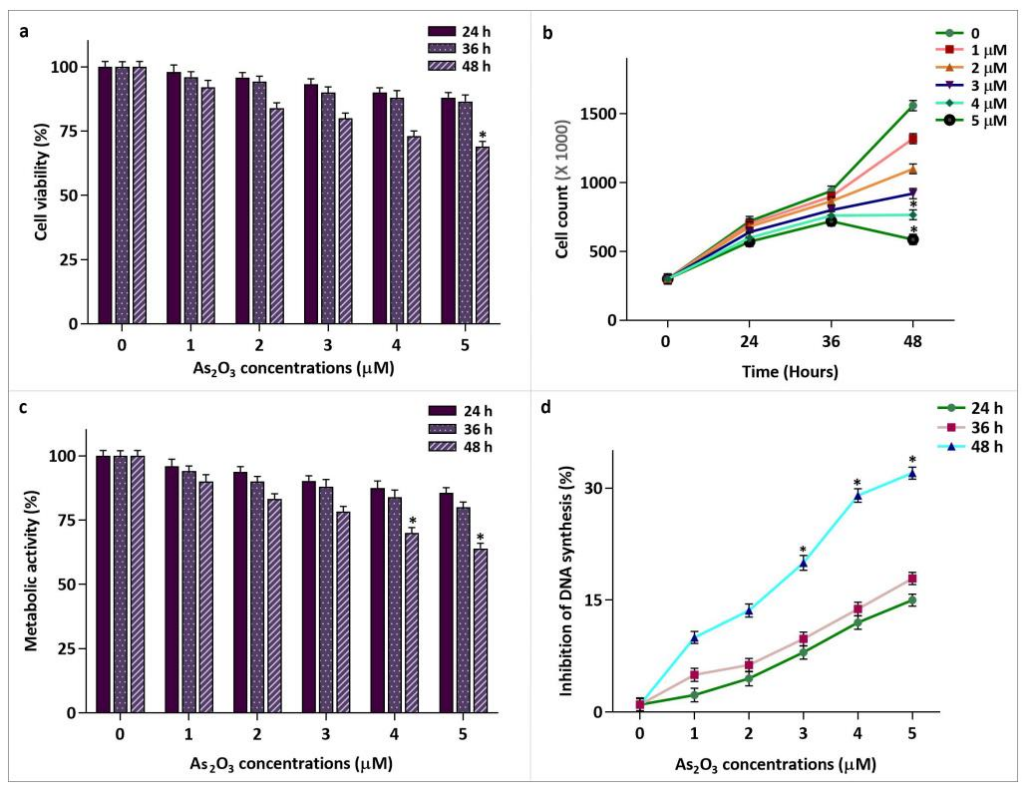
As2O3 induced cytotoxic and anti-proliferative effects on breast cancer cells. a and b: breast cancer-derived MCF-7 cells were treated with increasing concentrations of As2O3 (0-5 µM) up to 48 h. The results of trypan blue exclusion assay showed that As2O3 not only reduced cell survival in a dose- and time-dependent manner, but also decreased the number of MCF-7 cells; c: cells metabolic activity was inhibited upon treatment with As2O3; d: MCF-7 cells were incubated with the inhibitor up to 48 h and the suppressive effect on DNA synthesis rate was determined using BrdU cell proliferation assay. Values are given as mean ± SD of three independent experiments. *: P ≤ 0.05 represents significant changes from untreated control

To ascertain whether MCF-7 cells treatment with increasing concentrations of As_2_O_3_ (0-5 µM) up to 48 h could reduce the survival and proliferative rate of breast cancer cells, trypan blue, MTT, and BrdU cell proliferation assays were performed. As shown in figure 1, unlike 24 and 36 h treatment with As_2_O_3_ which had no significant effect on cell survival and growth kinetics, culturing cells for 48 h decreased the viability, cell count, and metabolic activity in a dose-dependent manner. Noteworthy, calculation of IC_50_ value for different methods of survival assessment after 48 h revealed that As_2_O_3_ is capable to reduce 50% of MCF-7 cell viability at the concentration of 8 µM (data not shown). The results of trypan blue and MTT assay were further substantiated by investigating the amount of DNA synthesis rate using BrdU incorporation assay which revealed that As_2_O_3_-plus-meltonin remarkably hampered the proliferative capacity of breast cancer cells. As presented in figure 1d, treatment of the cells at this point in time resulted in a significant inhibitory effect on DNA synthesis (nearly by 32%) at the concentration of 5 µM.

**Melatonin reinforced the cytotoxic effect of As2O3**

**Fig. 2:**
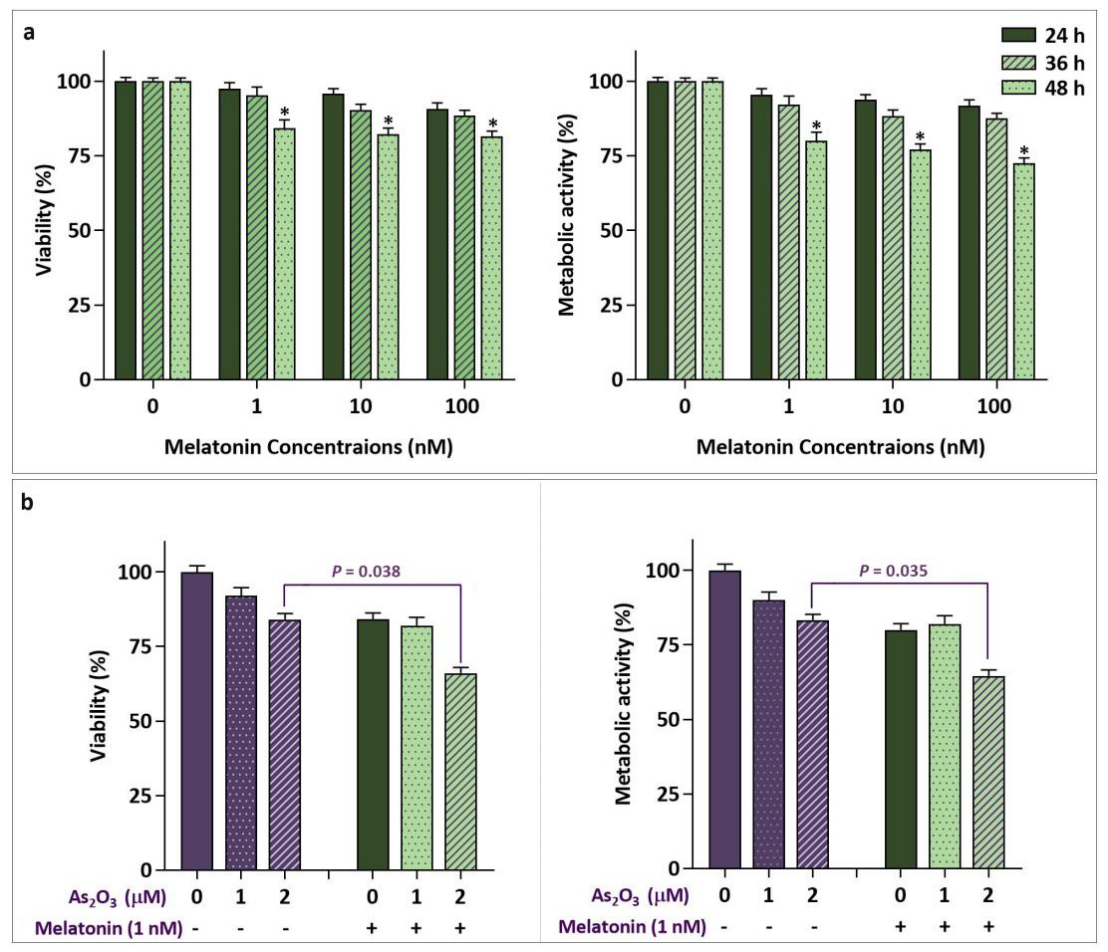
The synergistic effect of melatonin and As2O3. a: treatment of MCF-7 with increasing concentrations of melatonin (1-100 nM) up to 48 h resulted in the reduction of cell viability and metabolic activity; b: MCF-7 cells were treated with melatonin (1 nM) in combination with As2O3 (1 and 2 µM). The results of synergism evaluation experiments showed that melatonin could sensitize breast cancer cells to the anti-leukemic effect of As2O3. Values are given as mean ± SD of three independent experiments.*: P≤ 0.05 represents significant changes from untreated control

Given the IC_50_ value of As_2_O_3_ on breast cancer cells, which is considerably higher than its maximum dose used in clinical applications (17), we aimed to evaluate whether melatonin could enhance the effectiveness of As_2_O_3_ through reducing its effective concentration on MCF-7. Time- and dose- dependent experiments showed that melatonin alone exerted its cytotoxic and anti-proliferative effects only in a time- dependent manner. As depicted in figure 2a, 48 h exposure of MCF-7 cells to the lowest and highest concentrations of melatonin (1 nM and 100 nM) reduced cell viability by 16% and 19%, respectively. Therefore, we chose the physiological concentration of this indolamine (1 nM) for synergistic effect assessment. Interestingly, As_2_O_3 _in combination with melatonin reduced more effectively the number of MCF-7 viable cells (P=0.038) in comparison with either agents alone. This finding was further substantiated by MTT assay, showing that simultaneous treatment of cells with both agents efficiently hampered the metabolic activity (P=0.035) (Fig.2b). Determination of combination index (CI) and dose reduction index (DRI) values also clarified that melatonin boosted the cytotoxic and anti-proliferative effect of As_2_O_3_. Values of CI and DRI after 48 h treatment of MCF-7 cells are summarized in Table 2.

**Fig. 3:**
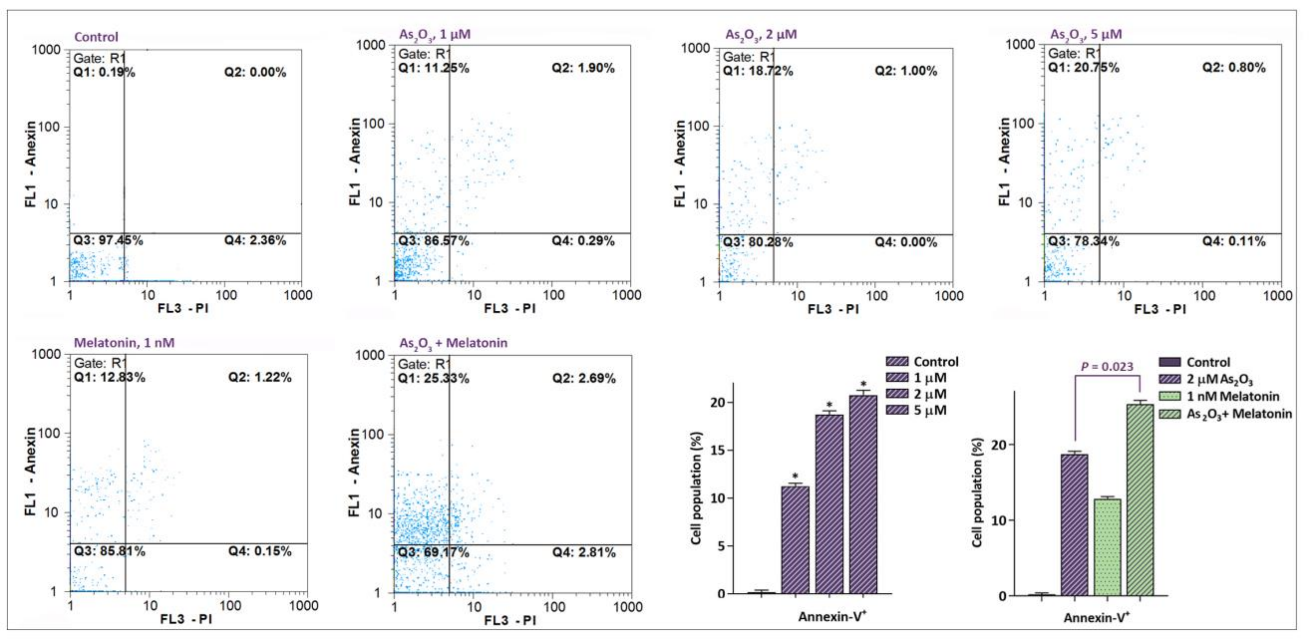
Treatment of MCF-7 cells with As2O3, either alone or in combination with melatonin, increased the percentage of apoptotic cells in MCF-7 cells. After incubation of breast cancer-derived MCF-7 cells with As2O3 and melatonin, the population of Annexin-V+ and Annexin-V/PI+ cells were investigated by using flow cytometry analysis. Values are given as mean ± SD of three independent experiments. *, P ≤ 0.05 represents significant changes from untreated control

**Fig. 4:**
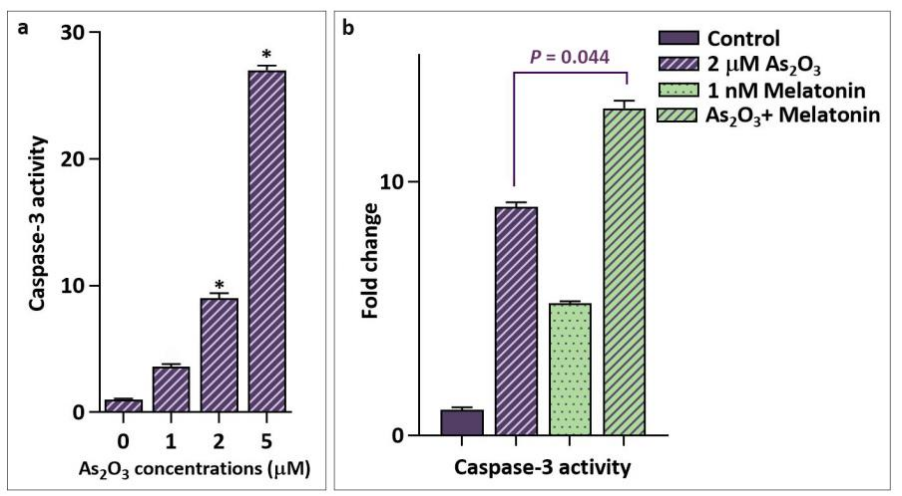
Activation of caspase-3-dependent apoptotic pathway in response to As2O3 and its combination with melatonin. a: As2O3 increased dose-dependently the enzymatic activity of caspase-3; b: a notable increase in caspase-3 activity was observed when MCF-7 cells were treated simultaneously with As2O3 and melatonin. Values are given as mean ± SD of three independent experiments. *: P ≤ 0.05 represents significant changes from untreated control

**Fig 5:**
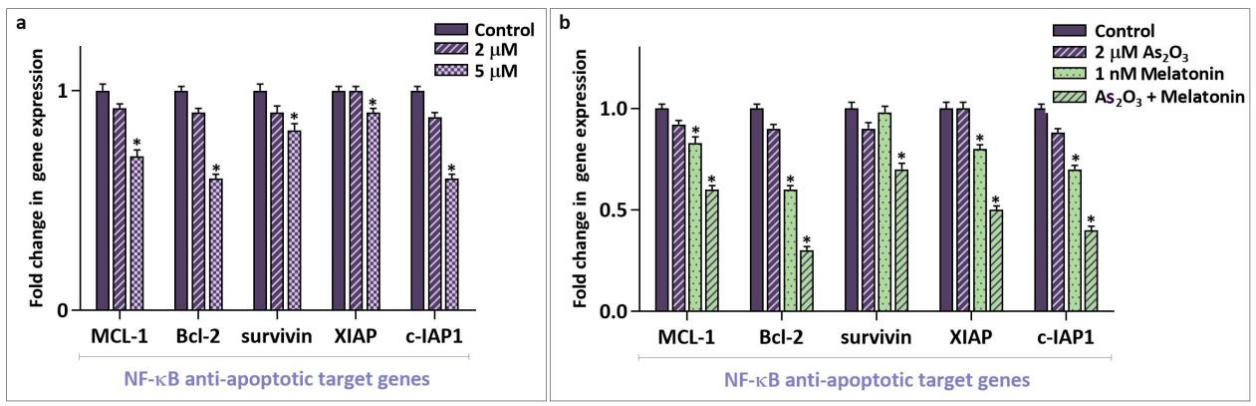
Effects of As2O3 alone or in combination with melatonin on the transcription of anti-apoptotic target genes of NF-κB in MCF-7 cells. a: the results of RQ-PCR analysis revealed that As2O3 at its highest concentration (5 µM) was able to reduce the mRNA level of different anti-apoptotic target genes of NF-κB in MCF-7 cells; b: combined treatment with melatonin and As2O3 potently reduced the mRNA expression level of NF-κB anti- apoptotic target genes. Values are given as mean ± SD of three independent experiments. *: P ≤ 0.05 represents significant changes from untreated control

**Table 1:** Nucleotide sequences of primers used for real-time RT-PCR

**Gene**	**Accession** **number**	**Forward primer (5′-3′)**	**Reverse primer (5′-3′)**	**Amplicon size (bp)**
***HPRT***	NM_000194	TGGACAGGACTGAACGTCTTG	CCAGCAGGTCAGCAAAGAATTTA	111
***BCL-2***	NM_000633	CGGTGGGGTCATGTGTGTG	CGGTTCAGGTACTCAGTCATCC	90
**Survivin**	NM_001168	CCAGATGACGACCCCATAGAG	TTGTTGGTTTCCTTTGCAATTTT	152
***MCL-1***	NM_021960	AGAAAGCTGCATCGAACCAT	CCAGCTCCTACTCCAGCAAC	183
***XIAP***	NM_001167	ATAGTGCCACGCAGTCTACAA	AGATGGCCTGTCTAAGGCAAA	101
***c-IAP1***	NM_001166	AGCACGATCTTGTCAGATTGG	GGCGGGGAAAGTTGAATATGTA	102

**Table 2:** Combination index (CI) and dose reduction index (DRI) for drug combination by As_2_O_3_ and melatonin

**As**_2_ **O**_3_		**Melatonin**		**CI**
**Concentration (µM)**	**DRI**	**Concentration (nM)**	**DRI**	
1	2.163	1	0.895	1.58
2	3.051	1	1.956	0.839

**Melatonin potentiated As**_2_ **O**_3_ **- induced apoptosis in MCF-7 cells**

To shed light on the mechanisms through which As_2_O_3_ induced its inhibitory effect on the survival rate of mammary adenocarcinoma cells, MCF-7 were exposed to escalated concentrations of the agent (1-5 µM) and the percentage of the annexin-V and annexin-V/PI positive cells were examined using annexin-V/PI staining assay. The results of flow cytometry analysis revealed that treating the cells with As_2_O_3_ increased the percentage of annexin-V positive cells. As presented in figure 3, the highest percentage of apoptotic MCF-7 cells was detected at the concentration of 5 µM, which elevated the proportion of annexin-V positive cells to 20.75%. Our finding was even noticeable in the synergistic effect assessment, where an enhanced induction of apoptosis was found using As_2_O_3_ in combination with melatonin. As depicted in this figure, 48 h treatment of cells with melatonin and As_2_O_3_ (2 µM) remarkably increased the percentage of annexin-V positive cells from 18.72% in As_2_O_3_- treated to 25.33% in melatonin/As_2_O_3_- treated group (P= 0.023), which indicated that melatonin at its physiological concentration is able to enhance the pro-apoptotic properties of As_2_O_3_.

**Stimulatory effect of melatonin on As2O3-induced apoptosis is mediated through caspase-3 activity**

In response to death stimuli, activated form of caspase-3 catalyzes the cleavage of many key cellular proteins, which eventually commit to cell apoptosis (18). To ascertain the involvement of caspase-3 activation in As_2_O_3_-induced apoptosis, we investigated the activity of the enzyme after treating MCF-7 cells with drug concentrations similar to those used for the annexin-V assay. Our results showed a concentration- dependent increase in the caspase -3 activity upon treatment with As_2_O_3_. Although treatment with 1 μM As_2_O_3 _did not induce significant enzymatic activity, exposing cells to 2 and 5 μM for 48 h increased caspase-3 activity by 9-and 27- fold, respectively (Fig. 4a). Noteworthy, co- treatment of the cells with melatonin and As_2_O_3_ also resulted in apoptotic cell death through a caspase-3-dependent cascade However, similar to annexin-V staining assay data, enzymatic activation of caspase-3 significantly increased when As_2_O_3_ was used in combination with melatonin (P =0.044) (Fig. 4b), substantiating the potentiating effect of melatonin for As_2_O_3_ treatment.

**Downregulation of NF-kB anti-apoptotic target genes following cell treatment with As2O3 and** **melatonin**

Conclusive evidence has stated that perturbed activation of NF-κB, a potent inducer of anti-apoptotic genes, contributes to the process of chemo-resistance in tumor cells (19). Our results revealed that while 2 µM As_2_O_3_ had minimal effect on NF-κB death repressor genes transcription (Fig.5a), physiological dose of melatonin robustly reinforced the suppressive effect of As_2_O_3_ on NF-κB downstream anti- apoptotic target genes. As illustrated in figure 5b, when MCF-7 cells were treated with both As_2_O_3 _and melatonin, the mRNA expression levels of *MCL-1*, *BCL-2*, survivin, *XIAP*, and *c-IAP1* reduced significantly (Fig. 5b).

## Discussion

During the ages, natural products have catered the basic needs of humans in the provision of medicines for the treatment of a broad spectrum of diseases (20). A traditional Chinese medicine As_2_O_3_ has long been of biomedical interest, and is largely investigated for treatment of a variety of cancers, ranging from hematological malignancies to solid tumors (21-23) However, its prodigious anti-cancerous efficacy has been rigorously overshadowed by the unfavorable side effects. In this regard, several strategies have been established thus far, to reinforce the cytotoxicity of this agent, especially for use in combination therapy. The recent discovery of the roles deviating from the canonical activities of melatonin has added a new perspective to reevaluate the application of this natural product of pineal gland in cancer therapeutic approaches (24). The results obtained in the present study showed that As_2_O_3_ reduced the survival and proliferative rate of MCF-7 cells and induced a caspase-3- dependent apoptotic pathway in this cell line. This finding was even more evident in the combined treatment, where we found that in the presence of melatonin the ability of As_2_O_3 _to decrease cell survival increased significantly in comparison with either drug alone. Consistently, previous studies conducted on breast cancer declared that melatonin may ameliorate the adriamycin-induced cardiac dysfunction through free radical scavenging or abrogating lipid peroxidation (25, 26). Additionally, it has been indicated that melatonin enhanced the apoptotic effect of the conventional chemotherapeutic drugs, such as tamoxifen (27), cis-platin (28), and 5‐fluorouracil (29). Although multiple lines of evidence have introduced melatonin as a promising candidate for adjuvant therapy (29, 31), further studies are now under way to characterize more precisely both the efficacy and the molecular mechanisms of action of this hormone in combination with anti-cancerous agents.

Conclusive evidence has stated that the activation of NF-κB, a potent inducer of anti-apoptotic genes, could be responsible for unexpected side effects of many commonly used chemotherapeutic drugs (19). Moreover, perturbation of NF-κB pathway in the pathogenesis of most human malignancies has attracted a great interest to the suppression of this nuclear transcription factor (32). Our study delineated that As_2_O_3_ at the concentration of 2 µM had minimal effect on the expression level of NF-κB target genes such as *MCL-1*, *BCL-2*, and the anti-apoptotic proteins of IAP family. Noteworthy, when As_2_O_3_ was used in combination with melatonin, its suppressive effect on the expression level of aforementioned genes turned to be more strengthened, indicative of the negative contributory role of melatonin on the transcription of anti-apoptotic target genes of NF-κB. This finding was in accordance with a study demonstrating that over-expression of *MCL-1*, a well-known member of anti-apoptotic genes of BCL-2 family, prevented the induction of apoptosis in multiple myeloma cells (33). The results of recent studies also revealed that suppression of either telomerase (34) or neurokinin-1 receptor (35) potentiated As_2_O_3_-induced cytotoxicity in APL-derived NB4 cells through down-regulating NF-κB anti-apoptotic target genes. In another study, Qiu et al. introduced asymmetric curcuminoid analogs as potent anticancer agents which not only reduced the survival of gastric cancer cells, but also enhanced the sensitivity of the cells to the common chemotherapeutic drug via downregulation of NF-κB activation (36). Moreover, it has been indicated that over-expression of anti-apoptotic proteins of BCL-2 family endows cancer cells a survival advantage (37-39). Herein, based on the suppressive effect of melatonin on the expression level of anti-apoptotic target genes of NF-κB, it is tempting to hazard a conjecture for the first time that probably the adjuvant effect of melatonin on the anti-tumoral activity of As_2_O_3_ is mediated through repressing the NF-κB activation. Consistently, the study by Lu et al. revealed that melatonin is capable of inhibiting nuclear translocation of NF-κB in lung cancer cells (40).

In conclusion, our results clearly demonstrated that besides the pro-apoptotic effect of As_2_O_3_ as a single agent in breast cancer, melatonin could significantly boost the anti-tumoral activity of this well-known chemotherapeutic drug in MCF-7 cells. Furtheremore, our results showed that melatonin-plus-As_2_O_3_ decreased the survival rate of breast cancer-derived MCF-7 cells by triggering a caspase-3-dependent apoptosis probably through suppressing the expression of anti-apoptotic target genes of NF-κB. Given the safety of melatonin, as the natural secretory product of pineal gland, the resulting data suggests that using melatonin in combination with As_2_O_3_ might be advantageous for expediting this hormone as a therapeutic agent in breast cancer.
